# SHISMA: SHape-driven inference of significant cell type-specific subnetworks from tiMe series single-cell trAnscriptomics

**DOI:** 10.1093/bioadv/vbag258

**Published:** 2026-09-03

**Authors:** Antonio Collesei, Pierangela Palmerini, Emilia Vigolo, Francesco Spinnato

**Affiliations:** Biostatistics and Bioinformatics, Veneto Institute of Oncology IOV-IRCCS, via Gattamelata 64, Padua, 35128, Italy; Immunology and Molecular Oncology Diagnostics, Veneto Institute of Oncology IOV-IRCCS, via Gattamelata 64, Padua, 35128, Italy; Immunology and Molecular Oncology Diagnostics, Veneto Institute of Oncology IOV-IRCCS, via Gattamelata 64, Padua, 35128, Italy; Department of Computer Science, University of Pisa, Largo Bruno Pontecorvo 3, Pisa, 56127, Italy

## Abstract

**Motivation:**

Recent advances in DNA and RNA sequencing technologies and the gradual decrease in costs have allowed to design serial experiments with timestamps, even at single cell resolution. This possibility unlocks a finer level of detail, as well as a huge amount of noisy information to decode. Tools inferring regulatory networks, or patterns, from this type of data often focus on trajectories, disregarding local shapes and fundamental time series primitives. Moreover, they fail to target the analysis on a few meaningful results, reporting large and noisy outputs that need further downstream analysis.

**Results:**

We describe SHISMA, a novel tool to infer significant cell type-specific co-dynamic gene subnetworks, from time series transcriptomic data, with strong statistical guarantees in terms of *P* value. SHISMA leverages isolated cell populations thanks to single-cell resolution, constructing cell type-specific pseudobulk time-series datasets. It then exploits a recently proposed time series primitive, the Bag-of-Receptive-Fields, adapted to discretize shorter temporal data and retain local shapes. SHISMA extracts significant groups of genes by performing a random walk approach on a protein–protein interaction network, with nodes identified by genes and scores derived from the shape-induced representation of the data, while properly validating via permutation and correcting for multiple hypothesis testing. Our extensive experimental evaluation on synthetic data shows that our tool is able to retrieve specific and significant subnetworks from time series transcriptomic data. Moreover, the subnetworks identified by SHISMA on real-world data confirm its ability to retrieve known cell type-specific processes, as well as potentially novel patterns and co-dynamic mechanisms.

**Availability and implementation:**

https://github.com/antoniocollesei/SHISMA

## 1. Introduction

In the last 15 years, the continuous advances in DNA and RNA sequencing technologies allowed both the collection of huge amounts of data and the development of methods to tackle an increasingly higher level of heterogeneity and resolution. Initially, the prohibitive costs forced researchers to model the temporal dimension through heterogeneity in *static* and observational datasets. This constraint led to the advancement of now-established bioinformatics fields, such as, among others, subclonal reconstruction (Caravagna *et al.* 2020, Tarabichi *et al.* 2021) and causal inference (Lecca 2021, Mansouri *et al.* 2022, Simionato *et al.* 2024), primarily for genomic variant data. The rise of single-cell RNA sequencing (scRNA-seq) technology enabled the possibility of revealing the transcriptional information at the cell level, with unprecedented resolution. To incorporate the time variable in scRNA-seq, the pseudo-temporal ordering (Reid and Wernisch 2016, Trapnell *et al.* 2014) was developed as a particularly novel approach. This computational method infers temporal trajectories (e.g. differentiation and development) by ordering cells on a maturation scale based on the expression of key genes. Concurrently, RNA velocity frameworks (La Manno *et al.* 2018, Bergen *et al.* 2020) emerged to infer directional single-cell dynamics over shorter timescales by modeling the mathematical kinetics of unspliced and spliced mRNA transcripts directly from individual unsynchronized cellular states. More recently, the gradual decrease in sequencing costs has unlocked the possibility to design serial scRNA-seq experiments over time to monitor transcriptional dynamics of samples (e.g. patients’ response to treatments) or evolution dynamics of cell cultures. This enabled the emergence of a novel data type called scRNA-seq time series, incorporating real, rather than pseudo-estimated, temporal information. In this context, two main trends emerged with the integration of the temporal label: refinement of trajectory reconstruction (Ding *et al.* 2022, Macnair *et al.* 2022), and inference of gene regulatory networks (GRNs) (Marku and Pancaldi 2023). Nevertheless, while trajectory algorithms build on the established concept of pseudotime, now being informed by the actual time label to refine the ordering of cells, GRN methods instead aim at reconstructing complex networks and cause-effect relationships between genes given their temporal dynamic. Within the latter field, algorithms such as Cardamom (Ventre 2021) and WASABI (Bonnaffoux *et al.* 2019) stand out by explicitly modeling transcriptional dynamics, regulatory waves, and causal propagation delays directly from real-time stamped scRNA-seq data matrices. Although the two approaches present interesting standpoints and challenges, they share two main limitations. First, they build upon established biological frameworks (i.e. pseudotime) rather than focusing on purely time-dependent inference. Second, they offer a holistic approach, usually computationally expensive, unable by design to produce targeted results, which is a critical aspect to translate computational findings into biological insights.

This work bypasses these limitations by introducing a different paradigm centered on co-dynamic subnetwork inference, leveraging core principles from pattern mining and time series learning theory. Rather than attempting to map complete directional causal circuits, like Cardamom and WASABI, SHISMA isolates localized, topologically connected functional groups of genes (subnetworks) within a protein–protein interaction (PPI) network that exhibit highly synchronized, identical temporal expression shapes across an absolute timeline. To achieve this, our workflow aggregates single-cell data upstream into cell type-specific pseudobulk profiles per timepoint, exploiting single-cell resolution to isolate cell populations without cellular composition biases. Crucially, we acknowledge that systemic discordance between mRNA and functional protein levels (often estimated to impact approximately 50% of genes due to complex post-transcriptional regulation) prevents direct linear abundance mapping. Therefore, SHISMA leverages the PPI database strictly as a topological structural constraint, narrowing the high-dimensional search space and suppressing spurious transcriptomic false positives by evaluating only elements with a topological constraint, offered by the PPI link. In Section 3.1, we present the procedure to obtain the absolute time series dataset from which SHISMA mines the shape-induced significant subnetworks. Methodologically, our algorithm leverages a promising but overlooked primitive in time series analysis: the Bag-of-Receptive-Fields (BoRF) transformation (Spinnato *et al.* 2024), derived from the more traditional Bag-of-Patterns (Baydogan *et al.* 2013). Analogous to the Bag-of-Words model in text analysis, the BoRF encodes a time series by counting the occurrences of discretized patterns. These patterns are extracted using a sliding window and capture local temporal structures, or *shapes*, within the time series. A further challenge we address is a chronic issue when working with large omics datasets: false positives. In our scenario, a false positive is a non-relevant subnetwork whose estimated *P* value is less than a confidence threshold α. This problem becomes more severe when performing inference from numerous parallel, short time series, requiring strict control of the family-wise error rate (FWER), which is the probability of outputting at least one false positive. In summary, our contributions are threefold. (i) We design SHISMA, a novel, translational algorithm to identify a list of significant gene subnetworks, guided by the local shape of time series subsequences specific to a cell type of interest, while controlling the FWER below a user-defined threshold. (ii) We show the need to correct for multiple hypothesis testing via network node permutation testing, as their associated SHAP scores serve as critical input to identify significant subnetworks. (iii) We perform an extensive evaluation on both synthetic and real-world scRNA-seq data in the specific biological domain of human immune response, to demonstrate the validity and robustness of our approach.

## 2 Methods

### 2.1 Background

Our algorithm integrates mathematical principles coming from a wide set of domains. For the sake of comprehension, we identified three main areas needing formalization and unification of their scientific notation. In the Supplementary Material we provide a summary of the terms used throughout the article to improve readability.

#### 2.1.1 Time series and BoRF s primitive

**Definition 1** (Time Series Dataset).

*A time series*, x*, is a sequence of T real-valued observations sampled over time*, $x=[x_{1},\ldots ,x_{T}]\in R^{T}$*. A time series dataset*, $X=\{x^{\left(1\right)},\ldots ,x^{\left(N\right)}\}\in R^{N\times T}$*, is a collection of N time series.*

**Definition 2** (Time Series Classification).

*Given a time series dataset* X  *and an associated label vector* $y\in {\left\{1,\ldots ,G\right\}}^{N}$*, where G denotes the number of distinct classes, the objective of Time Series Classification (TSC) is to learn a function f that assigns a predicted label* ${\hat{y}}^{\left(i\right)}$  *to each input time series* $x^{\left(i\right)}\in X$*, i.e.* $f(x^{\left(i\right)})={\hat{y}}^{\left(i\right)}$  *for all* i∈{1,…,N}*. The resulting predictions form the vector* $\hat{y}=[{\hat{y}}^{\left(1\right)},\ldots ,{\hat{y}}^{\left(N\right)}]\in {\left\{1,\ldots ,G\right\}}^{N}$.

Time series datasets can be used in a variety of tasks. This work exploits supervised learning, particularly TSC. In this setting, the central aim is to train a model *f* that generates predictions $\hat{y}$ that are as close as possible to the ground truth labels y. Many classification models additionally produce a probabilistic output $\hat{Y}\in {\left[0,1\right]}^{N\times G}$, representing class likelihoods, from which the final prediction $\hat{y}$ is obtained by selecting the most probable class for each instance. While achieving an acceptable level of classification accuracy is typically central, this work relies primarily on generating interpretable predictions that can provide insights into the model’s decision-making process, and concurrently unveil class-feature relationships. Since explanations are intended to clarify the behavior of the learned model, their practical usefulness depends both on the predictive reliability of the model and on the faithfulness and robustness of the explanations themselves (Crabbé and van der Schaar 2023). While recent work focuses on counterfactuals explainability (Wang *et al.* 2024), we leverage SHAP to attribute importance. To enable interpretability, time series can be transformed into tabular representations (Theissler *et al.* 2022). Among existing interpretable approaches, such as shapelets, interval-based, and dictionary-based, we focus on the latter, specifically on symbolic representations. Prominent methods include MrSEQL (Le Nguyen *et al.* 2019), MrSQM (Nguyen and Ifrim 2021), SCALE-BOSS-MR (Glenis and Vouros 2024), and BoRF (Spinnato *et al.* 2024), which achieves superior accuracy both in regular and irregular scenarios (Spinnato and Landi 2026). The BoRF extends the Bag-of-Patterns (Baydogan *et al.* 2013) by sliding strided, dilated windows over a time series to extract fields of length ℓ, potentially containing gaps. These patterns are standardized and discretized using SAX (Lin *et al.* 2007), which applies PAA (Keogh *et al.* 2001) to compute segment means, then quantizes them using Gaussian-distributed breakpoints into symbols.

**Definition 3** (BoRF).

*Given* $X\in R^{N\times T}$  *and H patterns, a BoRF is a matrix* $Z\in N^{N\times H}$*, where* $z_{h}^{\left(i\right)}$  *counts occurrences of hashed SAX pattern h in time series i.*

#### 2.1.2 Shapley values

The Shapley values, derived initially from the game theory domain (Shapley 1951), have gained significant popularity in explainable artificial intelligence research, since their modern formalization (Lundberg and Lee 2017). They offer a principled approach to feature attribution, aiming to fairly distribute a model’s prediction across its input features. The general formula to obtain feature attribution scores is provided by SHAP (SHapley Additive exPlanations), that extends Shapley values to model-agnostic explanation by using an additive surrogate model. More details can be found in Supplementary Material. Let *f* be the predictive model, *h* be the individual feature, and *H* be the set of input features. Then, the SHAP value for feature *h* is given by:


(1) $$w_{h}=\sum_{S\subseteq H\setminus \{h\}}\frac{|S|!(|H|-|S|-1)!}{|H|!}\left(f_{S\cup \{h\}}(z)-f_{S}(z)\right).$$

This equation captures the expected marginal contribution of feature *h* across all subsets S of the other features. Generally, computing exact SHAP values requires the investigation of, at least, $2^{H}$ subsets, resulting in exponential complexity, and therefore making it an NP−Hard problem, or even NP−Complete in some domains (Matsui and Matsui 2001). However, recent work focuses on model-constrained approximations to make SHAP computation polynomial in time, thus feasible in practice. For instance, in the tree-based models domains which is relevant to our work, it is sufficient to calculate SHAP values on a single tree, since the SHAP values of tree ensembles equal the sums of SHAP values of its individual trees, according to their additivity property (Lundberg *et al.* 2018). In this context, TreeSHAP (Lundberg *et al.* 2020) was developed as bottom-up Shapley value approximation method designed for tree-based models such as decision trees, random forests, and gradient-boosting trees. More recently, an even faster implementation has been developed, FastTreeSHAP (Yang, 2021), which achieves quicker calculations by optimizing the number of explored paths across the tree splits up to the leaf nodes. This is crucial when calculating contributions from an input dataset consisting of a high number of observations.

#### 2.1.3 Random walk with restart and hierarchical clustering

Biological processes are driven by intricate molecular interactions, often represented by networks such as PPI networks. In computational biology, they are typically modeled as network graphs N=(V,E,w), where vertices, or nodes, V correspond to biologically relevant entities (e.g. genes or proteins), edges E represent directed or undirected interactions between vertices, and weights $w:V\to R_{\geq 0}$ encode statistical or experimental evidence, in the form of scores. Hierarchical HotNet (Reyna *et al.* 2018) is a method designed to discover hierarchies of significantly altered subnetworks in a vertex-weighted biological network. The objective is to infer groups of genes, modeled as vertices in a PPI network, that are both highly altered and topologically connected in the underlying interaction network. To integrate the *a priori* knowledge, Hierarchical HotNet calculates a joint similarity matrix $P=(p_{ij})$ based on a random walk with restart. Given an adjacency matrix A and diagonal degree matrix $D=\text{diag}(\text{deg}(v_{1}),\ldots ,\text{deg}(v_{G}))$, the random walk transition matrix is $T=\beta {\left(I-(1-\beta )AD^{-1}\right)}^{-1}$, where β∈(0,1) is the restart probability within the random walk. The result is a similarity matrix incorporating both topological information and vertex scores, defined as $P=T\cdot \text{diag}(w(v_{1}),\ldots ,w(v_{G}))$. This approach penalizes high-degree nodes, while capturing indirect relationships through multi-step paths in the network. Exploiting the similarity matrix P, a family of directed graphs $H_{\delta }=(V,E_{\delta })$ is subsequently defined by setting a threshold δ: $E_{\delta }=\{(v_{j},v_{i})\in V\times V|m_{ij}\geq \delta \}$. By clustering strongly connected components (SCCs) of $H_{\delta }$, at each δ, a dendrogram is produced to capture relationships between clusters. This hierarchical approach allows the detection of subnetworks of different sizes. To compute the significance of the detected subnetworks, the algorithm compares the dendrogram to a null distribution generated by permutations of the node weights, while keeping their degree to preserve the network structure. Finally, a subnetwork is deemed altered if its cluster size exceeds what is expected under the null. For an in-depth description of the method, please refer to the original paper (Reyna *et al.* 2018). While SHISMA inherits this network propagation framework, we implemented a upgraded version that substitutes the directed SCC dendrogram assembly with a multi-scale percentile edge sweep, dense local topological weighting, and a direct permutation significance test (detailed in Section 2.2 and in supplementary data at Bioinformatics Advances online).

### 2.2 Algorithm

In this section, we present SHISMA, a novel method that identifies subnetworks of genes specific to a cell type of interest, driven by shapes, or subsequences, and significant (in terms of the *P* value), from scRNA-seq data, integrating biological domain knowledge through a PPI network. While doing so, we still control the FWER, given the high number of observations offered by scRNA-seq and a consequently higher amount of possible spurious links. Specifically, SHISMA takes in input a normalized dataset X, a genetic identity vector y, a cell type of interest $c_{r}$, a PPI-induced network N, and optionally a confidence level α, pattern length ℓ (<T), and an alphabet size *a*. Additionally, the user can choose the number of permutations $n_{p}$ and the dilation parameter *d*, which allows to control the spacing of the timepoints by skipping them and looking at broader structural patterns. In output, SHISMA produces a list of clusters, or subnetworks, ordered by their *P*-value, and pertaining to a specific pattern, or temporal shape. SHISMA’s pseudocode is available in Algorithm 1. Given the definitions in Section 2.1, our setting receives the following inputs: X is a list of *N* single cell transcriptomic time series, sampled at *T* timepoints, thus $x_{t}^{\left(i\right)}$ is the observed expression of gene $y^{\left(i\right)}$ at timestamp *t*; $c=[c^{\left(1\right)},\ldots ,c^{\left(N\right)}]\in {\left\{1,\ldots ,C\right\}}^{N}$ is the cell type related to time series $x^{\left(i\right)}$; N=(V,E) is the induced graph with V genes representing the nodes and E interactions derived from a PPI database representing the edges. SHISMA, whose graphical representation is shown in Fig. 1, and notation summary available in Supplementary, works in three steps, expanded in the following paragraphs.

Algorithm 1 SHISMA

**Input:** dataset X, genes y, cell types c, cell type of interest $c_{r}$, network N=(G,E), pattern length ℓ, alphabet size *a*, dilation *d*, confidence level α, permutations $n_{p}$, correction *K*

**Output:** ranked list of significant subnetworks $S_{p}$, with related shape pattern *h* and $p_{\mathrm{adj}}$

1: Z←BoRF(X,ℓ,d,a)

2: Z←(Z≠0)

3: $Z_{c_{r}},y_{c_{r}}\leftarrow \mathrm{filter}(Z,y,c=c_{r})$

4: $Y_{c_{r}}\leftarrow \mathrm{OneHotEncode}(y_{c_{r}})$

5: **for** Every pattern *h* in $Z_{c_{r}}$  **do** 

6: $T_{h}\leftarrow$fitTreeModel$\left(Y_{c_{r}},Z_{c_{r}}(h)\right)$

7:  **for** Every gene *y* **do** 

8:   $w_{yh}\leftarrow \mathrm{fastTreeSHAP}(T_{h},y)$

9:  **end for** 

10: **end for** 

11: $W=(w_{yh})$, output  ←∅

12: **for** every pattern *h* and score list $W_{h}$  **do** 

13:  $P\leftarrow \mathrm{RWRSimilarity}(N,W_{h})$

14:  S←clusteringSCC(P)

15:  $S_{p},p_{\mathrm{adj}}\leftarrow \mathrm{correctFWER}(S,n_{p},\alpha ,K)$

16:  $\mathrm{output}\leftarrow \mathrm{output}\cup \mathrm{annotate}(S_{p},h,p_{\mathrm{adj}})$

17: **end for** 

18: **return**output

**Figure 1:**
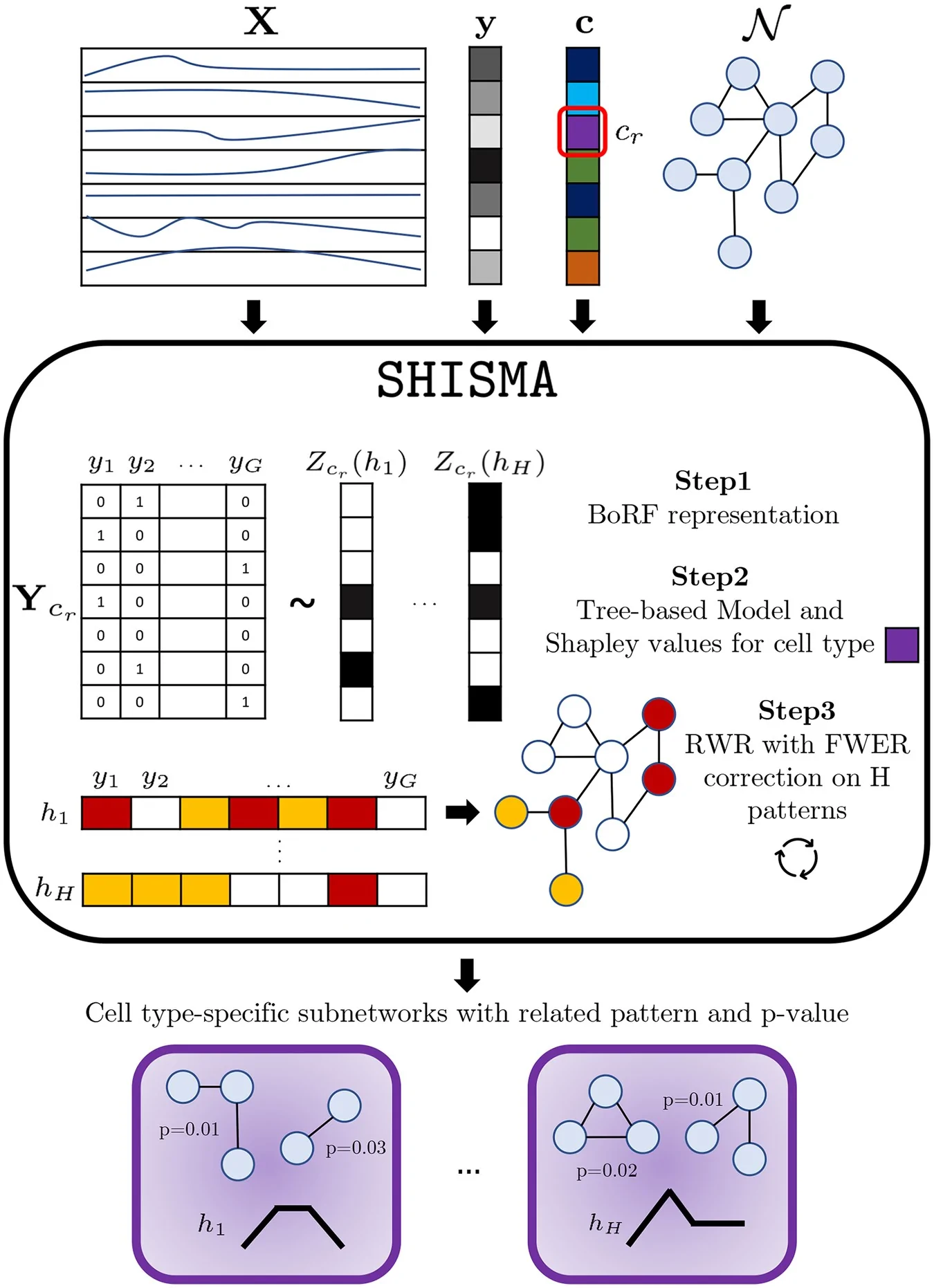
Graphical representation of our algorithm, SHISMA. At the top of the image, the main data inputs are described, including the time series dataset X, the gene identity array y, the cell identity array c, and the PPI-derived network N. In the box, the standard three-step workflow of SHISMA is described for cell type of interest $c_{r}$ represented by the color purple. At the bottom of the image, the results for $c_{r}$ are depicted in non-tabular form, with the main attributes being: pattern *h*, genes in the subnetwork, and corresponding *P* value.

### 2.3 Time series primitive transformation

The first step of our method consists in applying the BoRF function (Algorithm 1, line 1). As described in Section 2.1.1, the original dataset of time series X is converted into a representation space $Z\in N^{N\times H}$. In Z, features are represented by patterns *H*: informally, we can refer to these patterns as temporal shapes, since their main contribution is to characterize fixed-length trends of gene expression over time. The number of patterns depends on both the length ℓ and the alphabet size *a* used to encode the expression levels. Subsequently, we binarize Z to catch only the presence or absence of a specific pattern within the time series, and filter the information that is relevant to the cell type of interest $c_{r}$, thus yielding $Z_{c_{r}}$. As preparatory step to the following paragraph, we one-hot-encode the gene vector $y_{c_{r}}$ into a matrix $Y_{c_{r}}$.

### 2.4 Model fitting and score calculation

The second step consists of calculating gene score values via SHAP, as described in Section 2.1.2, for each pattern h∈H in $Z_{c_{r}}$. The feature attribution can only be achieved by fitting a compatible tree-based model for every *h*. The response variable is modeled with the following formula $Z_{c_{r}}(h)∼T_{h}(Y_{c_{r}})$, where $T_{h}$ is fitted as a binary class classification problem. Informally, at every iteration of the loop (Algorithm 1, lines 5–10), each gene’s contribution toward the prediction of the pattern’s presence is calculated, just as a feature’s importance is calculated in relation to an outcome of interest in traditional machine learning modeling. Thus, a cell type-specific dataset $W=(w_{yh})$ is yielded, corresponding to SHAP-based gene-pattern contribution scores.

### 2.5 Subnetworks computation

Finally, the last step consists in discovering cell type-specific, shape-induced subnetworks within N and determining their statistical significance. To do so, we significantly adapt and optimize the network propagation framework of Hierarchical HotNet (Reyna *et al.* 2018). Instead of evaluating system-wide directed graph component hierarchies, our framework exploits an undirected setting (given the undirected nature of the PPI edges) in synergy with a local topological density upgrade. First, edges in N are dynamically weighted based on the Jaccard similarity coefficient of their local neighborhoods to favor exchange within highly communicative communities. Second, rather than constructing a conventional dendrogram, SHISMA executes a multi-scale percentile search sweep (50th, 70th, 80th, 90th, 95th, 99th percentile) over an edge-score matrix computed via the outer product of the network-propagated gene scores. SCCs that emerge within strict biological size boundaries are collected as unique candidate subnetworks. To evaluate significance, we employ a non-parametric network permutation framework, generating $n_{p}$ randomized null networks by shuffling edge connections while preserving the degree distribution of the original graph. For each candidate subnetwork, discovered in the real graph, an empirical *P* value is calculated as the proportion of permuted iterations yielding a subnetwork of equal or greater statistical significance. Additionally, the algorithm is iterated for as many pattern lists *H* of scores are available, performing independent random walks with restart on the same network N. Thus, to address the severe multiple hypothesis testing burden, raw *P* values are adjusted using either false discovery rate (FDR) (Haynes 2013) or the more conservative Bonferroni. Finally, a cell type-based specificity filter, based on fold-change, is applied to retain subnetworks that are statistically relevant to the population under investigation. An in-depth description of this filter is available in Supplementary. The results are then parsed into a final report, indicating the shape, target cell type, subnetwork composition, and corrected *P* value.

## 3 Results

This section offers a deeper look at the results we obtained by deploying SHISMA. Section 3.1 introduces the real-world dataset and presents *in silico* findings by comparing with relevant biological literature. Section 3.2 assesses SHISMA’s stability under different circumstances by performing a set of tests on synthetic data. Section 3.3, finally, shows general performances related to runtime and justifies design choices in our pipeline.

### 3.1 Real-world experiments

We tested SHISMA on real-world data and evaluated the relevance of its findings. In the following paragraphs we will describe dataset preparation and the most significant results within the cell types of choice. Our findings highlight several biological processes reflecting responses to signals coming from both the exogenous stimuli provided to the culture system and the endogenous factors released from other immune populations.

#### 3.1.1 Experimental protocol and single-cell sequencing

The longitudinal single-cell dataset was generated from peripheral blood mononuclear cells isolated from eight healthy donors. Cells were initially primed with 1000 IU/ml of recombinant human interferon-γ (hIFN-γ), followed 24 hours later by activation with 50 ng/ml of functional-grade anti-CD3 antibody and 500 IU/ml of recombinant human interleukin-2 (rhIL-2). The culture medium was sequentially replenished with rhIL-2 at days 5, 9, and 12. To track the transcriptomic kinetics, scRNA-seq was performed across six distinct timepoints (days 0, 1, 2, 5, 9, and 15; T=6) spanning 48 total samples. Extended documentation regarding technical execution and downstream quality control filters is provided in Supplementary.

#### 3.1.2 Final dataset and experimental setup

Cell types were inferred with Azimuth (Hao *et al.* 2021), and subsequently manually refined by domain experts. As $c_{r}$ of interest, we chose to evaluate B cells: their role in the immune response is well-ascertained; therefore, they offer a good comparison baseline for our results. As a plus, their dynamics over time are transversal, offering a complete perspective on the immunological spectrum. Additionally, in Supplementary, we present insights on CD4 memory activated cells and CIK cells to offer a wider perspective on the immune landscape. To build the input list of time series X, we averaged expression across same cell types for each timepoint, and pivoted the gene-cell type matrix into a one-column array. We then joined timepoints by considering common *gene-cell type-patient* keys, thus recreating the *T*-timepoint time series. In our experimental setup, T=6, therefore the maximum length of patterns is shorter than *T*. As PPI-induced N, we consider two curated immunitary PPIs, Reactome and KW-0391 (https://string-db.org/), filtering out interactions with confidence score lower than 70% to ensure robustness. As additional input parameters, we then set pattern length ℓ spanning from 2 to 4 timepoints, alphabet size *a *= 3 (i.e. low, medium, high expression), $n_{p}$ = 1000, and dilation *d *= 1. Other hyperparameters are set as default, as BoRF relies on an internal heuristic that automatically defines a set of configurations from the characteristics of the dataset, in order to build a multi-resolution representation of the data. We report the most significant results in Table 1, obtained by correcting with Bonferroni.

**Table 1:** Subnetworks discovered by SHISMA on real-world datasets, focusing on B cells as $c_{r}$.^a^

ID	Pattern	Subnetwork	$P_{\text{adj}}$
** 2*a***	HMLH	RNF111, UBC, UBE2D1, UBE2D2, UBE2N, ZNRF2	.002
** 2*b***	MHML	ADA2, AMPD3, APRT, BTN3A1, BTN3A2, BTN3A3, CANT1, IL1F10, IL2, IMPDH1, IMPDH2, KPNA7, NME2, PKM, PNP, XDH	.002
** 2*c***	HLHH	CLTC, EEA1, GDI2, PIK3C3, RAB10, RAB14, RAB5B, RAB5C, RAB7A, RBSN, VAMP2	.003
** 2*d***	HHML	CALM3, CAMK2A, CAMK2B, CAMK2D, CAMK2G	.011
** 2*e***	LMHM	EIF4A3, PPIE, SNRPA1	.016
** 2*f***	HMLM	ARMC8, UBA1, UBA7, UBE2H, UBE2W, YPEL5	.028
** 2*g***	MHL	TRIM17, TRIM39, TRIM9, UBE2D1	.029
** 2*h***	LHLH	EEA1, GDI2, RAB5B, RAB5C	.035
** 2*i***	LHML	PJA1, UBE2D2, ZNRF2	.035
** 2*j***	LHMM	GDI2, LNPEP, RAB10, RAB14, RAB5B, RAB5C, RBSN, SEC22B, SNAP23, SNAP25, SNAP29, STX1A, STX3, STX4, VAMP2, VAMP3, VAMP7, VAMP8	.039
** 2*k***	HHML	ARMC8, UBE2H, YPEL5	.042
** 2*l***	HLHL	GDI2, RAB10, RAB14, RAB5B, RAB5C, RAB7A	.048

a Each resulting subnetwork is complemented with relative temporal pattern and sorted by statistical significance. The letters indicate low (L), medium (M), and high (H) expression. We report only the results with $P_{\text{adj}}$-value ≤.05.

#### 3.1.3 Results on B cells

Time-series analysis of the B-cell population revealed a temporal modulation across distinct subnetworks, reflecting a regulation of cell growth and aborted differentiation, antigen processing, and progressive population contraction. Cell growth and discouraged differentiation are reflected in subnets 2*e*, 2*b*, and 2*d*. In subnet 2*d* (Fig. 2), CAMK2D, involved in the maturation of B cells into plasma cells (Li *et al.* 2021), is progressively downregulated, suggesting unfavorable culture conditions for this cell type to promote differentiation. In the same subnet, CALM3 (Schmalzigaug *et al.* 2001) initially increases, advising a protective role of the cells from death, followed by a downregulation phase matching the progressive disappearing of B cells at later time points. In the initial survival phase, cell growth is supported also by genes involved in (i) mRNA splicing (SNRPA1, EIF4A3 and PPIE, subnet 2*e*), that are constantly increasing over time, (ii) nucleotide metabolism (ADA2, AMPD3, APRT, CANT1, IMPDH1/2, NME2, PNP, XDH, subnet 2*b*), and (iii) glycolysis (PKM, subnet 2*b*) (Yang *et al.* 2026). Antigen processing and class switching functions are actively regulated throughout the culture, captured by overlapping subnets (subnets 2*c*, 2*h*, 2*j*, and 2*l*). This functional maintenance is evidenced by a steady temporal increase in RAB GTPases (e.g. RAB7) (Runsala *et al.* 2023) and a fluctuating pattern driven by their regulator GDI2 (He *et al.* 2026), indicating an active control mechanism. Finally, progressive population contraction becomes dominant at later timepoints, as demonstrated by the robust upregulation of the ubiquitin-proteasome and protein catabolism machinery (subnets 2*a*, 2*f*, 2*g*, 2*i*, and 2*k*). The delayed peaking of these networks, driven by UBC (Bianchi *et al.* 2019), UBE2D1, E3 ubiquitin ligases of the TRIM family (Jawed *et al.* 2026), and the senescence regulator YPEL5 (Wu *et al.* 2025), indicates the onset of ubiquitin-mediated senescence, cellular degradation, and the ultimate competitive attrition of the B cells.

**Figure 2:**
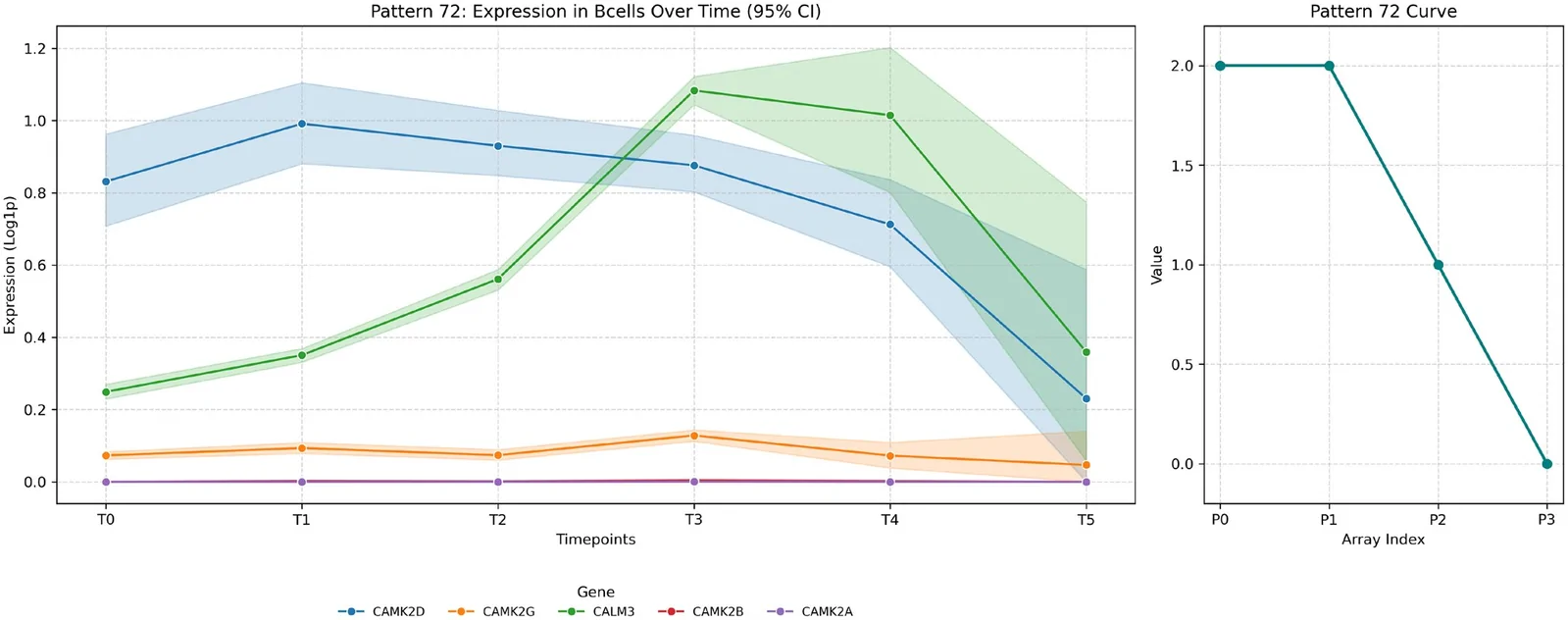
This graph serves as a representative example of the output generated for each significant subnetwork. Specifically, for subnetwork 2*d*, introduced in Section 3.1, the left panel displays the log-normalized expression of the component genes over the absolute timeline: mean value across patients is shown, with variance bounds. The right panel, instead, highlights the underlying driving pattern, where discrete values 0, 1, and 2 correspond to standardized low, medium, and high expression levels, respectively.

#### 3.1.4 Pathway enrichment and comparison with standard approaches

Since SHISMA’s perspective links unsupervised pattern mining with structural network propagation, it occupies a distinct niche from traditional active subnetwork discovery tools (Beisser *et al.* 2010, List *et al.* 2016). Therefore, a direct head-to-head architectural comparison is not feasible. Nonetheless, we performed a test to highlight differences between SHISMA and standard pathway enrichment methods applied to our real-world dataset. We compared the pathway enrichment profiles of our resulting subnetworks from B cell, CD4 memory activated T cell, and CIK cell data against a standard baseline bulk enrichment approach, relying on longitudinal differential expression via Analysis of Variance (ANOVA) to highlight highly-varying genes. Regarding B cells, the standard approach returned mostly systemic pathways (e.g. adaptive immune system and cytokine signaling) characterized by large gene sets, exhibiting significant background noise, and by the widespread presence of housekeeping genes, such as proteasome and ubiquitin-related subunits. In contrast, SHISMA effectively deconvoluted the larger network into highly specific, localized functional patterns with an average size of ∼7 genes, compared to ∼78 with the baseline method. By filtering out background noise, our method identified dynamic expression trends related to energetic adaptation (R-HSA-983712, R-HSA-382551), a progressive temporal increase in antigen processing (R-HSA-983168, R-HSA-983169, R-HSA-392499, R-HSA-8876198, R-HSA-9007101), and a gradual increase in lipid metabolism supporting cell maintenance (R-HSA-556833). These modules maintained robust statistical significance, demonstrating that our method provides a finer, and more complex resolution compared to traditional enrichment techniques. To quantitatively assess the agreement between SHISMA and the standard ANOVA approach in B cells, we computed the Jaccard similarity index (*J*) across both gene and pathway levels. Globally, the two methods exhibited modest overlap, sharing 84 significant pathways (J=0.153). Notably, SHISMA identified 358 unique pathways completely missed by standard enrichment, while ANOVA uniquely captured 107. When examining the top 100 pathways, ranked by significance, only nine pathways overlapped (J=0.047), including basic maintenance and immune cascades. Coherently, gene-level comparisons of the top 100 targets present J=0.031. Agreement metrics in B cells are mirrored by results in CIK cells, with extremely low *J* (0.07 for top 100 genes, 0.01 for top 100 pathways). Concerning CD4 memory activated T cells, ANOVA did not produce any significant result, confirming the limitations of its approach. Together, these overlap statistics quantitatively demonstrate that SHISMA provides extremely complementary insights. Rather than simply reproducing the broad transcriptomic patterns captured by ANOVA, SHISMA instead is able to successfully isolate specific, dynamic subnetwork interactions that otherwise remain invisible to standard pipelines.

Additionally, we decided to test a relevant tool working with single cell time series to infer GRN, LEAP (Specht and Li 2017). LEAP returns correlations between genes, considering potential gaps in the time series, with intent similar to the dilation parameter present in BoRF. Our test was designed not to serve as direct comparison with SHISMA, rather to pose a provocative challenge to GRN inference algorithms. LEAP was given a B cell-filtered real-world dataset, a subset of the dataset that we described in Section 3.1. The results present $10^{5}$ couples with an absolute correlation value over 99.5%; the $10^{6}$ most highly correlated couples fall above 93%. It appears evident how such an amount of *relevant* information poses a challenge in terms of follow up.

### 3.2 Synthetic evaluation

We assessed SHISMA’s performances in multiple ways. First, we tested the effectiveness of the multiple hypothesis testing correction employed by our algorithm to bound the FWER. To do so, we sampled datasets of variable size (N=[100,500,1000]), with an increasing number of time points (T=[6,8,10]), and with 5 or 10 patients, from a random distribution. For each experiment, a PPI with random edges was generated, still guaranteeing a minimum number of edges for each node to avoid a disconnected graph. This sampling ensures that no known relationships are present in the simulated datasets. The number of synthetic datasets for this experiment was 18, summing up to 1332 tested patterns (a=3, ℓ=[2,3,4]). We then ran SHISMA with standard parameters: our algorithm reports only two patterns as significant, consisting of a 0.15% failure rate. It is worth mentioning that both patterns were connected to the same synthetic dataset. The experiment states the importance of the multiple hypothesis correction routine, certifying the robustness against false positives. To submit additional proof, we ran the same experiment on a modified version of SHISMA, skipping the correction: for every tested dataset, at least one pattern was wrongly reported as significant.

Second, we confirmed the ability to mine gene-cell type hotspot relationships planted in a noisy dataset. In fact, a synthetic dataset drawn from a random distribution is fairly unrealistic, but served as mandatory sanity check. To prove SHISMA’s capabilities in a more practical scenario, we simulated log normalized pseudobulk data, planting an association between a group of selected genes (from *G000* to *G030*) and a selected cell type (*CT0*). We tested SHISMA’s ability to retrieve the selected genes as significant output. We acknowledged that the resulting recovered subnetworks corresponded exactly to the planted genes. This highlights SHISMA’s specificity, suggesting the production of reliable gene-cell type SHAP scores.

Lastly, exploiting the latter experimental setup, we performed an ablation study to justify the choice of the Decision Tree within SHISMA’s framework. We compared both performances and ability to retrieve the planted subnetworks of five models: the results are shown in Table 2. All models score high in terms of accuracy, but the metric we are particularly interested in is precision: in fact, the main objective is to retrieve true positives (i.e. genes associated with a pattern, and classified as such) with respect to false positives (i.e. genes wrongly classified as associated with a pattern). Decision Tree model stands out in terms of precision, while retaining a reasonable runtime and acceptable accuracy. However, the main discriminant is represented by its ability to retrieve exactly the planted genes as significant subnetwork, while other models fail to agree with the Decision Tree. The concordance between models in terms of top-scored genes is measured via Kendall coefficient (Kendall 1938) and presented in Supplementary.

**Table 2:** Comparison of classification models on accuracy, precision, recall, and runtime.^a^

Model	Accuracy	Precision	Recall	Time(s)
**Decision Tree**	0.83	**0.65**	0.16	3.46
**Random Forest**	**0.86**	0.61	**0.37**	9783.44
**Extra Trees**	**0.86**	0.59	0.29	13038.16
**Ridge**	**0.86**	0.59	0.29	5.45
**Logistic**	0.84	0.59	0.19	**1.99**

a The best result per individual metric is in bold.

### 3.3 Runtime and performance testing

We tested our method’s runtime under different inputs and parameters. We generated synthetic datasets as in Section 3.2, with the following characteristics: genes G=[100,500,1000,5000], timepoints T=[6,10,15], permutations $n_{p}=[10,100,500,1000]$, and planted timeseries equal to 10% of the total number of time series *N*. A constant number of cell types was considered (C=10). On the one hand, Fig. 3a shows the test outcome: an exponential increase in runtime can be noticed on datasets with thousands of genes. Premising that 5000 genes and 10 cell types yields a runtime of less than 10 minutes, the use of a less rich PPI (most of the ones publicly available are in the order of hundreds of genes) significantly reduces the runtime. Instead, the impact of timepoints does not affect runtime. On the other hand, Fig. 3b reiterates that the number of input genes constitutes the primary driver of execution time. Crucially, the runtime curves remain flat across the permutation axis: this near-constant behavior showcases the high efficiency of the underlying parallelized permutation framework. Both plots present the 95% confidence intervals, revealing that variance across varied patient and timepoint dimensions remains tightly bound, suggesting operational stability even under high dimensional stress.

**Figure 3:**
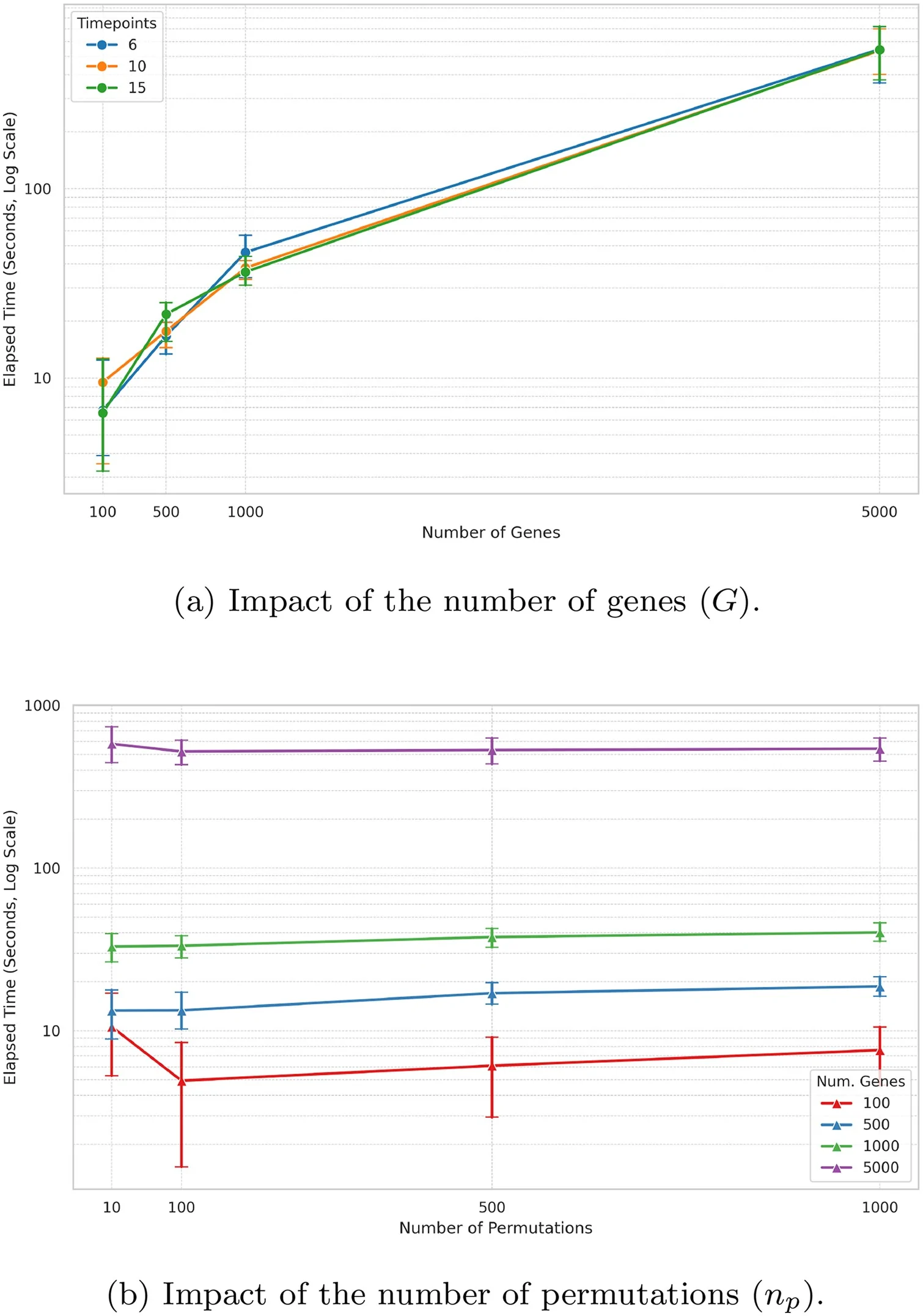
Runtime performance analysis of our method. (a) The top plot shows the effect of dataset size, linearly correlated with the number of genes, colored by number of timepoints. Execution time increases with the number of evaluated genes. (b) The bottom plot presents the effect of the number of permutations on runtime, colored by number of genes. Execution time remains unaltered by the number of permutations. Both plots offer error bars with 95% confidence intervals.

## 4 Discussion

In summary, the availability of time-resolved scRNA-seq data is a powerful instrument to potentially tackle the diversity and complexity of the immune landscape. Thanks to this technological evolution, we developed SHISMA, a robust framework for identifying cell type-specific subnetworks with coordinated temporal dynamics in longitudinal scRNA-seq data. By leveraging the BoRF representation, SHISMA captures local temporal shapes, while the integration of a tree-based feature attribution scoring and network topology analysis via random walk with restart, enables the discovery of statistically grounded gene modules.

Our design choice to exploit a shape-driven representation through BoRF allows for a degree of temporal awareness. However, SHISMA primarily detects changes in expression states rather than propagation delays, like WASABI or Cardamom. Moreover, while the underlying BoRF framework demonstrates state-of-the-art performance on irregular intervals without explicit temporal alignment, we acknowledge that treating highly irregular gaps as equidistant steps represents a simplification. Thus, a weighting framework to account for this remains an active area for future improvement. Another architectural constraint is our reliance on a Decision Tree. Although this restricts the model pool, it remains a necessary design choice to ensure the accuracy of our synthetic benchmarks, as discussed in Supplementary. Regarding statistical robustness, our algorithm employs an FWER-controlled framework by default to provide the highest confidence in discovered patterns. However, we also support flexibility in this regard: FDR-based correction suits real-world experimental settings, where the biological signal-to-noise ratio necessitates a balance between strict error control and sensitivity, while Bonferroni allows a more conservative approach. We provide this as a user-defined parameter to accommodate the diverse requirements of longitudinal datasets.

Nonetheless, pattern discovery on *short* time series comes with limitations. In fact, moral, feasibility and availability reasons preclude the possibility to have a reasonable number of time points: for instance, repeated biopsies in living patients need approval from an ethical committee. Thus, a current and arguably permanent shortcoming is posed by datasets with extremely short time series, such as T=2. By design, SHISMA slides a window of length ℓ for each subsequence across the time series, therefore it must hold ℓ<T to make the pattern discovery meaningful. Therefore, our algorithm is explicitly not recommended for minimal clinical designs restricted to simple pre- and post-treatment points where T<3. Instead, SHISMA is optimized as a specialized framework for complex longitudinal monitoring projects. In our experiments, we were able to sample the cell culture 6 times over 15 days; therefore, we pushed the discovery up to ℓ=4 and evaluated a richer set of patterns in our result section.

Additionally, we acknowledge that SHISMA exploits cell type assignments thanks to single-cell resolution, but methodologically operates on pseudobulk expression profiles. While this averaging step acts as a robust filter against single-cell stochasticity and dropout noise, it assumes a degree of unimodality within each cell type and may eventually mask intra-cluster heterogeneity or divergent sub-populations. We are working on an upgraded iteration of the framework that incorporates single-cell resolution directly, to resolve bifurcations and multi-stability. Consequently, the tool is best suited for identifying co-dynamic modules, rather than inferring directed interaction logic in a strictly causal manner.

Currently SHISMA exploits a cell type specificity filter that works similarly to differential expression analysis. However, this filter does not fully address another current limitation and possible extension: the possibility to recover differentially significant patterns, by comparing datasets sampled under diverse conditions. It could be extremely informative to evaluate the dynamics of a disease or a treatment, with respect to a control status. In fact, the final aim of our tool is not only to describe biological mechanisms, but also, and most importantly, to unveil potential treatment targets.

To this end, a future perspective sees the application of SHISMA to time series datasets of human tumors, as we believe it is robust enough to disentangle meaningful processes in such an intricate context. This would enhance knowledge and offer a novel therapeutic focus to tackle the disease with an unprecedented level of detail.

## Supplementary Material

vbag258_Supplementary_Data

## Data Availability

Code, instructions and data are available at GitHub https://github.com/antoniocollesei/SHISMA.
