# Supplementary material for: SHISMA: SHape-driven inference of significant cell type-specific subnetworks from tiMe series single-cell trAnscriptomics
Source: Bioinform Adv. 2026 Sep 3;6(1):vbag258. doi: 10.1093/bioadv/vbag258 (PMC13623514; doi:10.1093/bioadv/vbag258)
Supplement: Supplementary file 1 [file vbag258_supplementary_data.pdf]

Appendix

Shapley values

Linear Model Intuition

Shapley values are intuitively defined by means of a linear prediction model as follows. By considering the linear function:

$$\hat{f}(\mathbf{z}) = f(z_1, \dots, z_H) = \beta_0 + \beta_1 z_1 + \dots + \beta_H z_H, \tag{2}$$

where $\mathbf{z} \in \mathbb{R}^H$ is the input signal, and β_h represents the coefficient associated with feature h, the prediction is decomposed into a linear combination of contributions w_h of H set of features, as $g(\mathbf{z}') = w_0 + \sum_{h=1}^{H} w_h \mathbf{z}'_h$, where $\mathbf{z}' \in \{0,1\}^H$ indicates whether a feature is known ($\mathbf{z}'_h = 1$) or missing ($\mathbf{z}'_h = 0$). The contribution w_h of the h-th feature to the prediction $\hat{f}(\mathbf{z})$ is then given by:

$$w_h(\hat{f}) = \beta_h z_h - \mathbb{E}[\beta_h \mathbf{z}_{:h}] = \beta_h z_h - \beta_h \mathbb{E}[\mathbf{z}_{:h}], \tag{3}$$

where $\mathbb{E}[\mathbf{z}_{:h}]$ is the expectation of feature h over the respective background dataset column. By summing over all features, it is obtained:

$$\sum_{h=1}^{H} w_h(\hat{f}) = \hat{f}(\mathbf{z}) - \mathbb{E}[\hat{f}(\mathbf{Z})], \tag{4}$$

demonstrating that the sum of the attributions aligns with the difference between the prediction for the instance and the expected prediction.

Non-linear General Formula

However, the extension to non-linear models does not allow explicit coefficients. Therefore, SHAP (SHapley Additive exPlanations) extends Shapley values to model-agnostic explanation by using an additive surrogate model. Let f be the predictive model, and H be the set of input features. The model maps $\mathbf{z} \in \mathbb{R}^H$ to an output $f(\mathbf{z}) \in \mathbb{R}$ in regression, or $f(\mathbf{z}) \in [0,1]$ in binary classification. SHAP values are defined via a local surrogate model with w_0 as the *base value*, i.e., the default prediction when all features are removed. This surrogate model $g(\mathbf{z}')$ approximates $f(\mathbf{z})$ locally. SHAP values are the only unique solution of w_h that meet the three desired criteria of consistency, missingness, and local accuracy (Lundberg and Lee, 2017). To compute them, $f_{\mathbf{S}}(\mathbf{z})$ is defined as the model output when only a subset $\mathbf{S} \subset \mathbf{H}$ of the original features, $\mathbf{H}$, are present. Then, the SHAP value for feature h is given by:

$$w_h = \sum_{\mathbf{S} \subseteq \mathbf{H} \setminus \{h\}} \frac{|\mathbf{S}|!(|\mathbf{H}| - |\mathbf{S}| - 1)!}{|\mathbf{H}|!} \left(f_{\mathbf{S} \cup \{h\}}(\mathbf{z}) - f_{\mathbf{S}}(\mathbf{z}) \right). \tag{5}$$

This equation captures the expected marginal contribution of feature h across all subsets $\mathbf{S}$ of the other features. They then define

$$f_{\mathbf{S}}(\mathbf{z}) = \mathbb{E}_{\mathbf{z}_{\bar{\mathbf{S}}}}[f(\mathbf{z})]$$

as *marginal expectation*, where $\mathbf{z}_{\mathbf{S}}$ is the sub-vector of $\mathbf{z}$ restricted to the feature subset $\mathbf{S}$, and $\bar{\mathbf{S}} = \mathbf{H} \setminus \mathbf{S}$.

Random Walk with Restart (RWR) Methodology

Here we describe the formalization of the modified and expanded Hierarchical HotNet pipeline implemented within SHISMA (Reyna et al., 2018). Building directly upon the network-propagation concepts summarized in Section 2.1.3 and the high-level workflow outlined in the main text, this implementation introduces a localized topological density upgrade, a multi-scale percentile search space sweep, and a vectorized empirical permutation-testing routine coupled with False Discovery Rate (FDR) control (Haynes, 2013).

Topological Upgrade via Local Density-Weighted Adjacency

Let $\mathcal{N} = (\mathbf{V}, \mathbf{E})$ represent the input unweighted, undirected biological network, where $G = |\mathbf{V}|$ denotes the total number of distinct genes (nodes) in the graph backbone. To improve signal propagation through highly communicative, dense topological neighborhoods, we modify the traditional binary adjacency matrix $\mathbf{A}$ outlined in Section 2.1.3 by weighting active interactions using the Jaccard similarity coefficient of their local neighborhoods. For any interacting node pair $(u, v) \in \mathbf{E}$, the elements of the density-weighted adjacency matrix $\mathbf{A} \in \mathbb{R}^{G \times G}$ are defined as:

$$A_{uv} = \frac{|\Gamma(u) \cap \Gamma(v)|}{|\Gamma(u) \cup \Gamma(v)|} + \epsilon \tag{6}$$

where $\Gamma(u)$ denotes the set of immediate neighbors of node u in $\mathcal{N}$, and ϵ represents a minimal structural base weight ($\epsilon = 0.01$). This parameter ensures that sparse, linear path arrangements ("stringy edges") that possess zero shared neighbors remain marginally viable within the network. For non-interacting pairs $(u, v) \notin \mathbf{E}$, we define $A_{uv} = 0$.

The column-normalized transition matrix $\mathbf{W} \in \mathbb{R}^{G \times G}$ governs the step-wise directional distribution of the random walk and is computed via element-wise normalization over the active columns:

$$W_{uv} = \frac{A_{uv}}{\sum_{i=1}^{G} A_{iv}} \tag{7}$$

where columns with a sum of zero are forced to 1 to prevent numerical division-by-zero errors.

Closed-Form Topological Matrix Computation

As specified in the main text, computing the structural similarity profile via an iterative walk can be computationally intensive inside an interpretation framework. Given a fixed restart probability $\beta \in (0, 1)$ (where $\beta = 0.1$), we compute the closed-form global topological similarity matrix $\mathbf{P} \in \mathbb{R}^{G \times G}$ exactly once via direct matrix inversion:

$$\mathbf{P} = \beta \left(\mathbf{I} - (1 - \beta)\mathbf{W}\right)^{-1} \tag{8}$$

where $\mathbf{I} \in \mathbb{R}^{G \times G}$ represents the identity matrix. Because $\mathbf{P}$ is constructed on the density-weighted transition matrix $\mathbf{W}$, the network propagation step natively forces information to pool inside tightly interconnected topological cliques.

Multi-Scale Search Space Sweep

For each distinct temporal shape or pattern h, an initial feature attribution vector $\mathbf{f} \in \mathbb{R}^G$ is initialized using the absolute local surrogate model explanations (SHAP values) derived for the targeted celltype:

$$f_i = |w_{ih}| \quad \forall i \in \mathbf{V} \tag{9}$$

The network smoothing step projects these initial weights across the density-weighted graph structure to evaluate the integrated, smoothed gene score vector $\mathbf{s}_{\text{obs}} \in \mathbb{R}^G$:

$$\mathbf{s}_{\text{obs}} = \mathbf{Pf} \tag{10}$$

To locate structural subgraphs driven by these smoothed configurations without relying on an arbitrary, static threshold parameter, we implement a multi-scale search space sweep. First, an edge-score matrix $\mathbf{S}_{\text{edges\_obs}} \in \mathbb{R}^{G \times G}$ is constructed via the outer product of the smoothed node score vector, masked strictly by the unweighted, binary adjacency topology matrix $\mathbf{A}_{\text{binary}}$:

$$\mathbf{S}_{\text{edges\_obs}} = \mathbf{A}_{\text{binary}} \odot (\mathbf{s}_{\text{obs}} \cdot \mathbf{s}_{\text{obs}}^T) \tag{11}$$

where $\odot$ represents the Hadamard (element-wise) matrix product.

The pipeline evaluates network partitioning states across a set of edge-filtering percentiles $\delta = [50\text{th}, 70\text{th}, 80\text{th}, 90\text{th}, 95\text{th}, 99\text{th}]$. For each specific threshold scale δ_m, a sparse thresholded graph G_{δ_m} is generated by keeping only edges where $S_{\text{edges\_obs},\mu\nu} \geq \text{Percentile}(\mathbf{S}_{\text{edges\_obs}}, \delta_m)$. Connected components are extracted from G_{δ_m} and retained as valid candidate subnetworks if their structural size falls within strict boundaries:

$$\Omega = \{C \mid C \in \text{Components}(G_{\delta_m}),\ k_{\min} \leq |C| \leq k_{\max}\} \tag{12}$$

where the size filters $k_{\min} = 3$ and $k_{\max} = 30$ prevent the accumulation of uninformative small fragments or massive, uninterpretable global modules. Unique components discovered across all tested scales are aggregated into a unified candidate set Ω_{unique}.

Vectorized Permutation Evaluation and False Discovery Rate Control

To verify if an extracted candidate subnetwork component $C \in \Omega_{\text{unique}}$ displays internal network propagation scores that are significantly higher than random chance, we execute a highly optimized, vectorized empirical permutation test.

A randomized null attribution matrix $\mathbf{f}_{\text{perms}} \in \mathbb{R}^{G \times B}$ is instantiated by independently shuffling the rows of the observed vector $\mathbf{f}$ across $B = 1000$ permutations. The comprehensive empirical null score distribution space $\mathbf{s}_{\text{null}} \in \mathbb{R}^{G \times B}$ is evaluated in a single matrix multiplication operation:

$$\mathbf{s}_{\text{null}} = \mathbf{Pf}_{\text{perms}} \tag{13}$$

For each candidate component C, we evaluate its observed module statistic $M_{\text{obs}}(C)$ as the median value of its member nodes' smoothed scores:

$$M_{\text{obs}}(C) = \underset{i \in C}{\text{median}}\left(s_{\text{obs},i}\right) \tag{14}$$

An empirical null score vector $\mathbf{M}_{\text{null}}(C) \in \mathbb{R}^B$ is calculated identically across each permutation column index k:

$$M_{\text{null},k}(C) = \underset{i \in C}{\text{median}}\left(s_{\text{null},ik}\right) \tag{15}$$

The empirical p-value for the candidate component is derived as the proportion of random permutations where the null median score meets or exceeds the observed localized median statistic:

$$p(C) = \frac{\left(\sum_{k=1}^{B} \mathbb{I}\left(M_{\text{null},k}(C) \geq M_{\text{obs}}(C)\right)\right) + 1}{B + 1} \tag{16}$$

where $\mathbb{I}(\cdot)$ represents the standard indicator function.

As noted in the main text, running a multi-scale search sweep and evaluating H patterns create a multiple testing problem that inflates the rate of false positives. To control this, raw empirical p-values are adjusted globally per pattern using the Benjamini-Hochberg False Discovery Rate (FDR) procedure to yield final p_{adj}-values (Haynes, 2013):

$$p_{\text{adj}}(C) = \min_{j \geq i}\left(\frac{m \cdot H \cdot p_{(i)}}{i}\right) \tag{17}$$

where $p_{(1)} \leq p_{(2)} \leq \cdots \leq p_{(m)}$ represent the sorted empirical p-values, $m = |\Omega_{\text{unique}}|$ represents the total number of unique candidate subnetwork configurations generated across the sweep, and H the number of total evaluated patterns. Components are logged as statistically significant functional modules if they survive the final significance threshold ($p_{\text{adj}}(C) \leq \alpha$).

Celltype Specificity Filtering

A chronic challenge in celltype-specific omics inference is the recovery of ubiquitous and non-specific dynamics that manifest identically across the entire cellular ecosystem. To isolate modules uniquely synchronized within the target celltype c_r, SHISMA implements a downstream specificity filter. For each statistically significant subnetwork passing the global p_{adj} threshold α, we calculate its aggregate expression metrics. Let μ_{target} define the mean expression of the subnetwork's genes exclusively within the target celltype across the absolute timeline, and $\mu_{\text{background}}$ represent the mean expression of the same gene set across all other baseline celltypes in the dataset. A fold-change FC is computed as:

$$\text{FC} = \frac{\mu_{\text{target}}}{\mu_{\text{background}}} \tag{18}$$

To determine if the subnetwork's expression profile is significantly enriched in the target population, we evaluate the distribution of expression scores against the non-target background using a one-tailed statistical test (Wilcoxon rank-sum test or empirical label shuffling across celltypes), yielding a specific nominal value denoted as p_{spec}. Subnetworks failing to meet a strict specificity significance threshold (e.g., $p_{\text{spec}} < \alpha$) are discarded. This constraint guarantees that the output of SHISMA comprises modules that are not merely topologically tight and temporally distinct, but strictly localized to the specific celltype under investigation.

Synthetic Experiments

Ablation Study on Model of Choice

Following the ablation experiment presented in Section 3.2, we measured the agreement on the 30 top-scored genes according to a set of classifiers: Decision Tree, Random Forest, Extra Trees, Ridge, and Logistic. As already mentioned, Decision Tree scored at the top the 30 planted genes, being the obvious choice; as a pairwise comparison between models, we measured the Kendall coefficient between the lists of top-scored genes, showing affinity between Decision Tree and the Logistic.

**Model**	Decision Tree	Random Forest	Extra Trees	Ridge	Logistic
Decision Tree	1.0	0.25	0.11	0.33	**0.61**
Random Forest	0.25	1.0	0.27	0.10	0.24
Extra Trees	0.11	0.28	1.0	0.05	0.20
Ridge	0.33	0.10	0.06	1.0	0.44
Logistic	**0.61**	0.24	0.20	0.44	1.0

Table 3. Pairwise agreement matrix among the evaluated classification models. The diagonal represents the maximum agreement between lists of top-scored genes (Kendall coefficient = 1.0), while off-diagonal values indicate the extent of agreement between each pair of models.

Real-World Experiments

Cell culture and single-cell RNA sequencing

Fresh peripheral blood samples were collected from 8 healthy donors. Peripheral blood mononuclear cells (PBMCs) were then isolated and cultured with the addition of 1000 IU/mL of recombinant human interferon-γ (hINF-γ). After 24 hours, 50 ng/mL of CD3 pure-functional grade human antibody (Miltenyi) and 500 IU/mL of recombinant human interleukin 2 (rhIL2) (Proleukin, Novartis) were added to the culture. Cell medium was replaced with the addition of 500 IU/mL of rhIL-2 at day 5, 9, and 12. scRNA-seq was performed at day 0, 1, 2, 5, 9 and 15 (T=6) for a total of 48 samples. 4 samples from different donors were multiplexed with the 3' CellPlex Kit Set A using Cell Multiplexing Oligo (CMO) and Dual Index Kit NN Set A, according to manufacturer's protocol (10X Genomics). The targeted cell recovery was estimated at 30000 cells (7500 cells for each sample). cDNA libraries were obtained with the Chromium Next GEM Single cell 3' Reagents v3.1 and Dual Index TT Set A kits, following manufacturer's instructions (10X Genomics). Libraries were sequenced in two batches of 4 donors on a NovaSeq6000 (Illumina) at 20000 read pairs per cell for the 3' Gene Expression Libraries and 5000 read pairs per cell for the Cell Multiplexing Libraries. More details regarding quality control can be found in Section 5.4.2.

Quality Control

Raw data from scRNA-seq in FASTQ format were processed and mapped to the human reference genome (GRCh38) with Cell Ranger software (10x Genomics) to generate feature-barcode matrices, using default parameters. Samples were demultiplexed using Cell Ranger multi pipeline to assign cell barcodes to their respective sample origin. Resulting gene and data were analyzed with R package Seurat v5. Cell doublets were detected with scDoubletFinder and filtered out. Pooled distribution of the following quality control (QC) metrics was assessed for the 8 donors within each timepoint: UMI counts, unique gene counts, and percentage of mitochondrial (mt) and ribosomal (ribo) DNA. Low-quality cells were filtered using standard QC conditions for blood-derived immune populations and adjusted per timepoint. To account for batch effects, the Harmony algorithm was used. Dimensionality reduction, clustering and celltype identification, and Principal component analysis (PCA) were performed. For cluster identification, a shared nearest neighbor (SNN) modularity graph was constructed. Clustering analysis was performed using Louvain algorithm implemented in the FindClusters function, with a resolution parameter of 0.71. Celltype annotation was initially performed using automatic annotation through Azimuth reference database followed by manual expert-guided refinement.

Pathway enrichment results

Enrichment analysis of SHISMA results on B cells revealed three primary biological themes among the top 20 significant pathways (Table 4). First, fluctuating gene expression within the ATPase proton pump pathway (R-HSA-983712) reflects dynamic adaptation to energetic demands, supporting cell proliferation and function previously described. Second, antigen processing pathways, driven by both ubiquitin-proteasome and RAB-GDI2 mechanisms, showed a progressive increase over time (R-HSA-983168, R-HSA-983169, R-HSA-392499, R-HSA-8876198, R-HSA-9007101), confirming the active functional state of B cells already discussed in Section 3.1. Finally, a gradual temporal increase in lipid metabolism (R-HSA-556833) further supports cell maintenance, as reported in previous studies (Kubo et al., 2023; Shinton et al., 2022). To provide a comprehensive overview, CD4 Memory Activated T cells and CIK cells underwent the same comparative analysis, yielding the results presented in Table 5 and Table 6, respectively. In CIK cells, the enrichment analysis highlighted a strong representation of metabolic and nutrient-sensing pathways, such as insulin receptor recycling, amino acid regulation, and the cellular response to starvation. This reflects the intense energetic demands and metabolic reprogramming required to sustain their activation and robust proliferation (Klein Geltink et al., 2018). Conversely, the results for CD4 Memory Activated T cells revealed a significant overrepresentation of RNA polymerase pathways, underscoring the massive and immediate transcriptional burst characteristic of memory T cell reactivation. Since memory T cells are called to a rapid recall, this heightened polymerase activity drives the accelerated synthesis of ribosomes, tRNAs, and mRNAs, enabling them to deploy effector cytokines and execution machinery significantly faster than naive cells (Macian et al., 2002).

**Enriched Pathway**	**Adjusted P-val**
Amino Acids Regulate mTORC1	1.35e-47
Neddylation	2.31e-19
RAB GEFs Exchange GTP For GDP On RABs	2.88e-12
Translocation Of SLC2A4 (GLUT4) To Plasma Membrane	9.60e-12
Metabolism Of Lipids	1.70e-11
Membrane Trafficking	1.73e-11
IL-6-type Cytokine Receptor Ligand Interactions	2.79e-10
EPHB-mediated Forward Signaling	2.83e-10
Phase 0 - Rapid Depolarisation	1.78e-09
Glycosphingolipid Metabolism	5.71e-09
Metabolism Of Nucleotides	2.32e-08
Metabolism Of Carbohydrates	8.67e-07
Butyrophilin (BTN) Family Interactions	2.24e-06
mRNA Splicing	5.88e-06
TICAM1, RIP1-mediated IKK Complex Recruitment	1.25e-05
Synthesis Of Active Ubiquitin: Roles Of E1 And E2 Enzym	8.44e-05
Class B/2 (Secretin Family Receptors)	9.81e-05
Acyl Chain Remodelling Of PE	1.12e-04
Translocation Of ZAP-70 To Immunological Synapse	2.16e-03
LDL Clearance	2.72e-03

Table 4. Top-20 unique and significantly-enriched pathways related to B cell real-world results, presented in Section 3.1.

Results on CD4 memory activated cells

CD4 memory activated T cell population were defined by the expression of typical memory cell surface markers, as extensively reported in the literature (Gustafson et al. 2022). In this specific context, the addition of cytokines during the ex vivo culture expansion procedure drives their antigen (Ag)-independent activation (Cano-Gamez et al., 2022). This cell population emerges at day (D) 5 of culture and gradually decreases overtime until D15. When SHISMA was tested, the analysis revealed several gene subnetworks related to cell cycle control, vesicle trafficking, and protein translation whose modulation remained stable over time, indicating the maintenance of a CD4+ memory T cell steady state in culture (subnetworks 7*b*, 7*d*, and 7*e*). In contrast, subnetworks associated with immune cell activation showed reduced expression over time. In particular the transcription factor STAT1 was identified in 7*a* (comprising RNF19B, STAT1, and UBE2L6) and in subnetwork 7*c* (comprising DDX58, IFNAR1, IL6, ISG15, and STAT1) both of which relate to immune cell activation in response to IFN-γ stimuli (Peng et al., 2025). STAT1 is typically triggered by INF-γ and, in combination with STAT4, induces TBET which initiates the signaling cascade promoting the differentiation of naive CD4+ T cells toward a memory phenotype (Whitmire, 2014). Although both subnetworks of genes remain active, their overall expression decrease represents broader decline in memory T cell activation. Overall, these results demonstrate that while initial ex vivo stimuli successfully primed the cells, the absence of further specific signals drives a gradual decline of their activation program together with a maintenance of the cellular steady state.

Results on Cytokine-Induced Killer cells

We next focus on a peculiar immune cell population named Cytokine-Induced Killer (CIK) cells, which share functional and phenotypic properties of both T and Natural Killer (NK) cells, and are identified by the co-expression of both CD3 and CD56 (NCAM1) molecules (Cappuzzello et al., 2023). The analysis revealed several significant subnetworks related to cell survival and anti-apoptotic pathways (subnetwork 7*c*), active lipid metabolism (subnetwork 7*b*), and vesicle trafficking (subnetworks 7*a* and

Enriched Pathway	Adjusted P-val
Insulin Receptor Recycling	6.76e-51
Transferrin Endocytosis And Recycling	6.76e-51
ROS And RNS Production In Phagocytes	1.26e-43
Iron Uptake And Transport	7.78e-42
Ion Channel Transport	1.20e-39
Signaling By Insulin Receptor	6.91e-38
Amino Acids Regulate mTORC1	4.45e-37
O-linked Glycosylation Of Mucins	1.10e-35
Termination Of O-glycan Biosynthesis	1.10e-35
Defective C1GALT1C1 Causes TNPS	1.10e-35
Defective GALNT12 Causes CRCS1	1.10e-35
Defective GALNT3 Causes HFTC	1.10e-35
Cellular Response To Starvation	3.68e-35
Diseases Associated With O-glycosylation Of Proteins	1.25e-34
O-linked Glycosylation	1.25e-34
RHO GTPases Activate WASPs And WAVEs	7.47e-34
Diseases Of Glycosylation	3.35e-30
Dectin-2 Family	4.69e-29
Regulation Of Actin Dynamics For Phagocytic Cup Formation	8.53e-29
FCGR3A-mediated Phagocytosis	8.53e-29

Table 5. Top-20 unique and significantly-enriched pathways related to CD4 Memory Activated T cell real-world results, presented in Section 3.1.

Enriched Pathway	Adjusted P-val
Insulin Receptor Recycling	6.76e-51
Transferrin Endocytosis And Recycling	6.76e-51
ROS And RNS Production In Phagocytes	1.26e-43
Iron Uptake And Transport	7.78e-42
Ion Channel Transport	1.20e-39
Amino Acids Regulate mTORC1	4.35e-39
Signaling By Insulin Receptor	6.91e-38
RNA Polymerase III Chain Elongation	3.47e-37
RNA Polymerase III Transcription Initiation From Type 1 Promoter	3.47e-37
RNA Polymerase III Transcription Initiation From Type 2 Promoter	3.47e-37
RNA Polymerase III Transcription Termination	3.47e-37
Cellular Response To Starvation	4.62e-37
RNA Polymerase III Abortive And Retractive Initiation	3.37e-36
RNA Polymerase III Transcription Initiation	3.37e-36
RNA Polymerase III Transcription Initiation From Type 3 Promoter	3.37e-36
RHO GTPases Activate WASPs And WAVEs	7.47e-34
Regulation Of Actin Dynamics For Phagocytic Cup Formation	8.53e-29
FCGR3A-mediated Phagocytosis	8.53e-29
Transport Of Small Molecules	1.31e-27
Fcgamma Receptor (FCGR) Dependent Phagocytosis	6.02e-25

Table 6. Top-20 unique and significantly-enriched pathways related to CIK cell real-world results, presented in Section 3.1.

7d). The active modulation and sustained expression of genes involved in these mechanisms are sign of an active cellular state, in concordance with the proliferation of the CIK cell population observed over time, particularly from D5 to D15 of culture. Among these significant networks, subnetwork *7e* is of particular interest as it includes key genes belonging to the TNF signaling pathway. Notably, this subnetwork includes TNFRSF1A, a receptor widely known to trigger downstream pro-inflammatory and pro-survival NF-κB pathways in T cells (Webb et al., 2016), alongside the ligand TNFSF15 and the decoy receptor TNFRSF6B, both of which are critical in balancing pro-survival and pro-apoptotic signaling in T cells maturation and differentiation (Richard et al., 2015; Jin et al., 2013). In the context of CIK cell culture, the sustained or dynamic modulation of this specific axis likely provides the crucial survival signals necessary to sustain their prolonged ex vivo expansion and to promote their cytotoxic activity. Overall, the results obtained from SHISMA analysis confirm the sustained pro-survival metabolism of the CIK cell population, directly reflecting the effects of external stimuli aimed at driving their ex vivo expansion.

ID	Pattern	Subnetwork	p_{adj}
7*a*	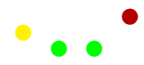	RNF19B, STAT1, UBE2L6	0.002
7*b*	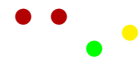	BTN3A1, BTN3A2, BTN3A3, XDH	0.007
7*c*	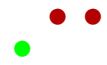	DDX58, IFNAR1, IL6, ISG15, STAT1	0.011
7*d*	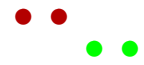	DDX3X, EIF4A1, EIF4A2, EIF4A3, EIF4E, EIF4E2, EIF4E3, EIF4G1, EIF4G2, EIF4G3	0.012
7*e*	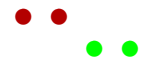	ATP6AP2, ATP6V0A1, ATP6V0A2, ATP6V0A4, ATP6V0B, ATP6V0C, ATP6V0D1, ATP6V0D2, ATP6V0E1, ATP6V0E2, ATP6V1A, ATP6V1B1, ATP6V1B2, ATP6V1C1, ATP6V1C2, ATP6V1D, ATP6V1E1, ATP6V1E2, ATP6V1F, ATP6V1G1, ATP6V1G2, ATP6V1G3, ATP6V1H, TCIRG1	0.016

Table 7. Subnetworks discovered by `SHISMA` on real-world datasets, focusing on CD4 memory effector cells as c_r. Bonferroni correction has been applied.

ID	Pattern	Subnetwork	p_{adj}
8*a*	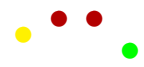	GDI2, LNPEP, RAB10, RAB14, SNAP23, STX4, VAMP2	0.002
8*b*	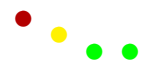	APOB, CYB5R3, HBB, HP, SAR1B, SEC24B, SURF4	0.002
8*c*	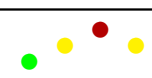	BCL2, BCL2L1, CD74, CPPED1, CST3, CSTB, CTSB, CTSD, CTSH, CTSL, CTSS, LGMN	0.004
8*d*	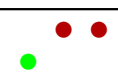	COPB1, GDI2, LNPEP, RAB10, RAB14, SAR1B, SEC13, SEC22B, SEC23A, SEC24A, SEC24B, SEC24C, SEC24D, SEC31A, SNAP23, SNAP25, SNAP29, STX1A, STX3, STX4, VAMP2, VAMP3, VAMP8	0.008
8*e*		TNFRSF1A, TNFRSF6B, TNFSF15	0.043

Table 8. Subnetworks discovered by `SHISMA` on real-world datasets, focusing on CIK cells as c_r. Bonferroni correction has been applied.

Summary of Notation used in `SHISMA`

Notation	Description
Time Series and Data Representation	
$\mathbf{X}, \mathbf{x}, x_t$	Time series dataset, a single time series, and an observation at time t
N	Number of time series in the dataset
T	Length of a time series (number of time points)
$\mathbf{y}, \hat{\mathbf{y}}$	Ground truth and predicted gene identity vectors
G	Number of distinct genes (classes)
$\mathbf{c}, c_r$	Vector of celltype labels and the celltype of interest
C	Number of distinct celltypes
Bag-of-Receptive-Fields (BoRF) and Model Explanation	
ℓ, a, d	Pattern length, alphabet size and dilation parameter for SAX discretization
h, H	An individual pattern (feature) and the total set of patterns
$\mathbf{Z}$	Bag-of-Receptive-Fields feature matrix ($\mathbb{N}^{N \times H}$)
$f, \mathcal{T}$	A general classification function and a fitted tree-based model
w_{yh}	SHAP score for the contribution of pattern h to gene y
$\mathbf{W}$	Matrix of scores
α, K, n_p	Significance level, correction method, and number of permutations
Network and Subnetwork Analysis	
$\mathcal{N}$	Graph representing the PPI network, $\mathcal{N} = (\mathbf{V}, \mathbf{E})$
$\mathbf{V}, \mathbf{E}$	Set of vertices (genes) and edges (interactions) in the network
$\mathbf{A}, \mathbf{D}$	Adjacency and diagonal degree matrices of the network
β	Restart probability for the random walk
$\mathbf{P}$	Joint similarity matrix derived from the random walk with restart

Table 9. Summary of notation.
